# Trends in the Management of Headache Disorders in US Emergency Departments: Analysis of 2007–2018 National Hospital Ambulatory Medical Care Survey Data

**DOI:** 10.3390/jcm11051401

**Published:** 2022-03-03

**Authors:** Seonkyeong Yang, Yulia Orlova, Abigale Lipe, Macy Boren, Juan M. Hincapie-Castillo, Haesuk Park, Ching-Yuan Chang, Debbie L. Wilson, Lauren Adkins, Wei-Hsuan Lo-Ciganic

**Affiliations:** 1 Department of Pharmaceutical Outcomes & Policy, College of Pharmacy, University of Florida, Gainesville, FL 32611, USA; yang.se@ufl.edu (S.Y.); hpark@cop.ufl.edu (H.P.); c.chang@ufl.edu (C.-Y.C.); debbie.wilson@ufl.edu (D.L.W.); 2 Neurology Department, College of Medicine, University of Florida, Gainesville, FL 32611, USA; yulia.orlova@neurology.ufl.edu; 3 College of Pharmacy, University of Florida, Gainesville, FL 32611, USA; a.lipe@ufl.edu (A.L.); macy.boren@ufl.edu (M.B.); 4 Department of Epidemiology, Gillings School of Global Public Health, University of North Carolina at Chapel Hill, Chapel Hill, NC 27599, USA; jhincapie-castillo@unc.edu; 5 Center for Drug Evaluation and Safety, College of Pharmacy, University of Florida, Gainesville, FL 32611, USA; 6 Health Science Center Libraries, University of Florida, Gainesville, FL 32611, USA; lauren.adkins@ufl.edu

**Keywords:** headache, primary headache, migraine, NHAMCS, ED visits, neuroimaging, opioid analgesic, triptan, antiemetic, trend

## Abstract

We examined trends in management of headache disorders in United States (US) emergency department (ED) visits. We conducted a cross-sectional study using 2007–2018 National Hospital Ambulatory Medical Care Survey data. We included adult patient visits (≥18 years) with a primary ED discharge diagnosis of headache. We classified headache medications by pharmacological group: opioids, butalbital, ergot alkaloids/triptans, acetaminophen/nonsteroidal anti-inflammatory drugs (NSAIDs), antiemetics, diphenhydramine, corticosteroids, and intravenous fluids. To obtain reliable estimates, we aggregated data into three time periods: 2007–2010, 2011–2014, and 2015–2018. Using multivariable logistic regression, we examined medication, neuroimaging, and outpatient referral trends, separately. Among headache-related ED visits, opioid use decreased from 54.1% in 2007–2010 to 28.3% in 2015–2018 (P*_trend_* < 0.001). There were statistically significant increasing trends in acetaminophen/NSAIDs, diphenhydramine, and corticosteroids use (all P*_trend_* < 0.001). Changes in butalbital (6.4%), ergot alkaloid/triptan (4.7%), antiemetic (59.2% in 2015–2018), and neuroimaging (37.3%) use over time were insignificant. Headache-related ED visits with outpatient referral for follow-up increased slightly from 73.3% in 2007–2010 to 79.7% in 2015–2018 (P*_trend_* = 0.02). Reflecting evidence-based guideline recommendations for headache management, opioid use substantially decreased from 2007 to 2018 among US headache-related ED visits. Future studies are warranted to identify strategies to promote evidence-based treatment for headaches (e.g., sumatriptan, dexamethasone) and appropriate outpatient referral and reduce unnecessary neuroimaging orders in EDs.

## 1. Introduction

Headache disorders are among the most common neurological disorders, with a worldwide prevalence of 46% in the adult population [1]. Severe and sudden headaches often lead patients to seek emergency medical attention, accounting for 3.5 million emergency department (ED) visits per year in the United States (US) [2], with most attributed to primary headache disorders [3]. According to the International Classification of Headache Disorders 3rd edition (ICHD-3), common types of primary headaches include migraine, tension-type headaches, and trigeminal autonomic cephalalgias (e.g., cluster headaches) [4].

Patients with primary headache disorders often receive unnecessary neuroimaging or medications with low-quality evidence during ED visits due to the difficulty in differentiating primary headaches from secondary headaches, incomplete relevant medical histories, and a lack of consensus on the best treatment strategies in ED settings. The priority in headache management in the ED is to identify potentially life-threatening conditions [5], and thus neuroimaging can be ordered for patients with red flags for secondary headaches [6,7]. Due to multiple reasons that lead to overuse of imaging in ED settings (i.e., fear of missing a serious diagnosis, preventing potential medical malpractice, patient demands, financial incentives) [8], patients with primary headache disorders may undergo unnecessary neuroimaging despite guidelines discouraging routine neuroimaging [9,10,11,12,13]. Selective and judicious neuroimaging is required to avoid unnecessary radiation exposure and mitigate health care costs.

Once secondary headaches are ruled out through neurologic examination and diagnostic evaluation, the goal of primary headache treatment in the ED focuses on immediate symptom relief and functional improvement. The 2016 American Headache Society (AHS)’s acute migraine treatment guidelines in ED settings recommend the use of intravenous metoclopramide, intravenous prochlorperazine, and subcutaneous sumatriptan as first-line treatments and parenteral dexamethasone to prevent recurrence [14]. Although these medications have been mainly studied among patients with migraine, other types of primary headaches respond well to these medications [15,16]. The AHS guidelines do not recommend other parenteral medications, including opioid analgesics, diphenhydramine, lidocaine, and octreotide, as first-line treatment of primary headaches in ED due to limited clinical evidence on their efficacy [14]. Current evidence has particularly cautioned against the use of opioids to treat primary headaches due to their lack of efficacy [17,18,19], the risk of developing medication overuse headaches [20,21,22,23], impeding responsiveness to other acute treatments [24,25], and other adverse outcomes (e.g., abuse and addition) [26]. Furthermore, opioid use among patients with migraine in ED settings increases the risk of a prolonged ED stay and recurrent ED visits [27,28,29]. Given that multiple factors may contribute to acute headache treatment, we aimed to examine the trends and patient and visit characteristics of headache management in the ED from 2007 to 2018 in the US.

## 2. Materials and Methods

### 2.1. Study Design and Data Sources

This cross-sectional study used the ED component of the National Hospital Ambulatory Medical Care Survey (NHAMCS) data from 2007 to 2018, which contains nationally representative samples of US-hospital-based ambulatory care settings. The publicly available NHAMCS data are collected and distributed by the US National Center for Health Statistics (NCHS) [30]. NHAMCS randomly selects a representative sample of ED visits using a three-stage probability sampling design. Geographically defined areas, hospitals, and ED service areas are randomly selected sequentially in each stage. Annually, approximately 500 nationally representative noninstitutional, general, and short-stay hospitals (excluding federal, military, and Veterans Administration hospitals) in the US participate in NHAMCS. During a 4-week randomly assigned reporting period within each calendar year, data are recorded in each sampled ED using questionnaires for a random sample of visits [31]. This study was deemed exempt from review by the University of Florida Institutional Review Board.

### 2.2. Study Cohort

In the primary analysis, we identified visits for adult patients (≥18 years) with a primary ED discharge diagnosis of headaches (hereafter headache-related ED visits). We identified headache disorders (including migraine, tension-type headache, trigeminal autonomic cephalgia, or not otherwise specified (NOS) headache) using the International Classification of Diseases, Ninth and Tenth Revisions, Clinical Modification (ICD-9-CM/ICD-10-CM) codes (Table S1). The rationale for including only headaches listed as a primary ED discharge diagnosis was to exclude potential secondary headaches for which the management focuses on the underlying medical condition, not the headache.

### 2.3. Main Outcomes Measures

Among headache-related ED visits, we examined three types of outcomes, including (1) medications given in the ED or prescribed at ED discharge, (2) neuroimaging use including computed tomography (CT) and magnetic resonance imaging (MRI) of the head, and (3) outpatient referral for follow-up. We used generic equivalent codes in the Multum Lexicon Plus^®^ system to identify medications of interest in NHAMCS (Table S2). For the trend analysis, we classified medications into 8 therapeutic classes: (1) opioid analgesics, (2) butalbital, (3) ergot alkaloids/triptans, (4) acetaminophen/nonsteroidal anti-inflammatory drugs (NSAIDs), (5) antiemetics, (6) diphenhydramine, (7) corticosteroids, and (8) intravenous (IV) fluids. We excluded opioid antitussives classified as ‘124: antitussives’ or ‘132: upper respiratory combinations’ by the Multum Lexicon Plus^®^. Ergot alkaloids and triptans are migraine-specific therapies that share similar mechanisms of action; thus, we examined them in a group. Antiemetics included dopamine receptor antagonists and 5-HT3 receptor antagonists. Although diphenhydramine also has an antiemetic property, it is more likely to be used to prevent an adverse effect of dopamine receptor antagonists (i.e., akathisia) in headache-related ED visits. Therefore, we examined diphenhydramine separately. We classified combination products using the following hierarchy order based on their analgesic potency: opioids > butalbital > ergot alkaloids/triptans > acetaminophen/NSAIDs > diphenhydramine. IV fluid use was identified by a separate variable available in the NHAMCS data. Data collection for the number of medications prescribed, supplied, administered, or continued varied across years (i.e., up to 8 medications prior to 2011, 12 medications in 2012–2013, and up to 30 medications since 2014). To ensure findings were comparable across years, the primary analysis was restricted to the first 8 medication codes listed. The latter is consistent with previously published literature using NHAMCS data [32]. We separately reported the numbers of medications given in the ED and prescribed at discharge. When sample sizes permitted it, we also analyzed the most commonly used individual medications and treatment patterns (e.g., monotherapy or combinations) among headache-related ED visits. Furthermore, we examined coadministration patterns of diphenhydramine and dopamine receptor antagonists among headache-related ED visits. We identified neuroimaging use of either head CT or head MRI among headache-related ED visits. We also identified whether a patient had an outpatient referral for follow-up using available data in NHAMCS.

### 2.4. Patient and Hospital Characteristics 

The patient characteristics of interest included age group (18–34, 35–49, 50–64, and ≥65 years), sex (female and male), race (White and non-White), payment source for the visit (Medicare, Medicaid, commercial insurance, and others), the number of chronic conditions (0, 1, and ≥2), and several important comorbidities of headaches, including cardiovascular diseases and depression status. The number of chronic conditions, cardiovascular diseases, and depression status were identified using separate variables available in NHAMCS, not using the diagnosis variables. NHAMCS reported pain intensity by category (e.g., none, mild, moderate, severe) prior to 2009, and with a pain scale (e.g., from 0 to 10) starting in 2009. To be consistent across years, we categorized pain intensity as none (0), mild (1–3), moderate (4–6), and severe (7–10) based on the literature [33]. The hospital characteristics of interest included type of providers who provided services during each ED visit (ED physician, consulting physician, ED resident/intern, nurse practitioner, and physician assistant), geographic region (Northeast, Midwest, South, and West), and whether the hospital was located in a metropolitan or non-metropolitan area.

### 2.5. Statistical Analysis

As recommended by the NCHS, we used the survey procedures (i.e., PROC SURVEYFREQ, PROC SURVEYLOGISTIC) that account for complex survey design and sampling weights of NHAMCS to obtain national estimates with accurate standard errors [34]. The NCHS does not recommend reporting results when the unweighted number for a given variable is less than 30 or the relative standard error is greater than 30% due to their unreliability [35]. To obtain reliable national estimates with sufficient sample sizes, we aggregated annual data into 3 time periods (i.e., 2007–2010, 2011–2014, 2015–2018). To examine the characteristics for different headaches, we stratified analyses by subtype of headache: migraine versus NOS headaches. We compared the characteristic differences between two groups using standardized mean difference (SMD), where SMD > 0.1 indicates non-negligible differences [36]. We conducted separate multivariable logistic regression analyses to test the significance of trends in the use of headache medications, neuroimaging, and outpatient referral for follow-up among headache-related ED visits over time, adjusting for age, sex, race, payment source, and practice region.

To ensure the robustness of our findings, we conducted four sensitivity analyses: (1) including all available medication codes (i.e., up to 8 medications between 2007 and 2011, up to 12 medications in 2012 and 2013, and up to 30 medications beginning in 2014); (2) including all available diagnosis codes listed (i.e., up to 3 diagnosis codes between 2007 and 2013 and up to 5 diagnosis codes beginning in 2014); (3) including all available medication codes and diagnosis codes listed for each patient visit during study periods; and (4) excluding visits having any medical conditions potentially associated with secondary headaches (e.g., subarachnoid hemorrhage (SAH), post-traumatic headache, brain tumor; Table S3) among all available diagnosis codes, visits resulting in hospital admission, and visits ending in death. Two-sided p-values less than 0.05 were considered statistically significant. All analyses were conducted with SAS version 9.4 (SAS Institute Inc, Cary, NC, USA).

## 3. Results

### 3.1. Patient Characteristics between 2015 and 2018

Out of 33 million headache-related ED visits from 2007 to 2018, two-thirds (63.9%) were due to NOS headaches and one-third (32.9%) to migraines. Tension-type headaches and trigeminal autonomic cephalalgias only represented 3.2% of headache-related ED visits. Headache-related ED visits slightly increased from 27.1/1000 visits in 2007–2010 to 29.0/1000 visits in 2011–2014, and then decreased to 24.1/1000 visits in 2015–2018 (P*_trend_* = 0.002; Figure S1). Table 1 shows the patient characteristics of headache-related ED visits categorized by migraine and NOS headaches during 2015–2018. Most headache-related ED visits were from patients aged <50 years (70.7%), female (72.9%), and White (70.3%). Nearly half of headache-related ED visits were from patients without any chronic diseases (46.3%). Cardiovascular diseases and depression were present in 32.2% and 13.5% of headache-related ED visits, respectively. More than half of headache-related ED visits were associated with severe pain based on the patient-reported pain scale (53.0%). The most common payment sources for headache-related ED visits were commercial insurance (31.3%) and Medicaid (29.5%), followed by Medicare (16.3%). Three or more medications were administered in half of headache-related ED visits (53.5%), while no medication was prescribed at ED discharge among half of the headache–related ED visits (54.1%). Most headache-related ED visits (85.7%) were overseen by ED physicians, and approximately 6% were overseen by consulting physicians. Headache-related ED visits were most prevalent in the Southern region (38.3%) and metropolitan areas (86.2%).

Compared to NOS-headache-related visits (Table 1), migraine-related visits were more likely to be from patients aged <50 years (78.1% versus 66.5%), females (82.0% versus 68.0%), Whites (82.2% versus 64.7%), those with depression (17.5% versus 12.1%), and those with severe pain (65.0% versus 47.3%). NOS-headache-related visits were more likely to be from patients with cardiovascular diseases (36.0% versus 25.5%) (all SMD > 0.1). The most common payment source for migraine-related visits and NOS-headache-related visits were commercial insurance (35.7% and 29.2%) and Medicaid (30.6% and 29.4%). Having ≥3 medications administered in the ED was more common for migraine-related visits in comparison to NOS-headache-related visits (67.6% versus 46.1%). Migraine-related visits were more likely to occur in metropolitan areas compared to NOS-headache-related visits (88.5% versus 81.2%).

### 3.2. Trends in Medication Use, Neuroimaging Use, and Outpatient Referral for Follow-Up between 2007 and 2018

**Figure 1:**
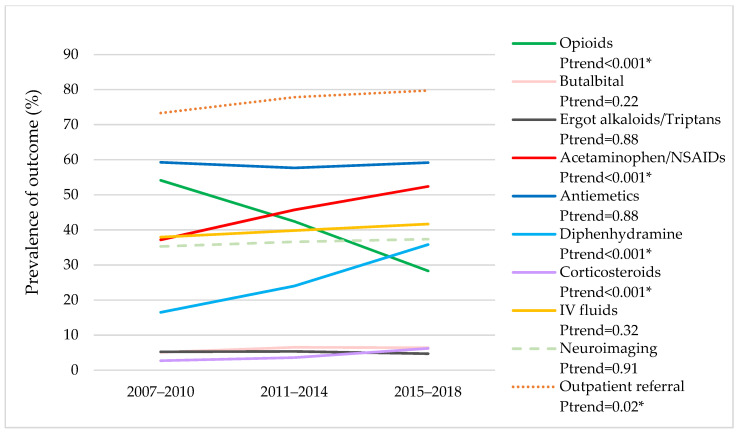
Trends in medication use, neuroimaging use, and referrals to follow-up among headache-related ED visits: 2007 to 2018 NHAMCS data. Abbreviations: ED: Emergency Department; NHAMCS: National Hospital Ambulatory Medical Care Survey; NSAIDs: nonsteroidal anti-inflammatory drugs; IV: intravenous. * A statistically significant trend with P*_trend_* < 0.001. All P*_trend_* were adjusted for age, sex, race, payment source, and practice region.

Figure 1 summarizes the adjusted trends in medication use, neuroimaging use, and outpatient referral for follow-up among headache-related ED visits. Opioid analgesic use decreased by half from 54.1% in 2007–2010 to 28.3% in 2015–2018 among headache-related ED visits (P*_trend_* < 0.001). Conversely, increased trends were observed in the use of acetaminophen/NSAIDs (37.2% to 52.4%, P*_trend_* < 0.001), diphenhydramine (16.5% to 35.8%, P*_trend_* < 0.001), and corticosteroids (2.7% to 6.2%, P*_trend_* < 0.001) from 2007–2010 to 2015–2018. The use of butalbital (6.4% in 2015–2018, P*_trend_* = 0.22), ergot alkaloids/triptans (4.7%, P*_trend_* = 0.88), and antiemetics (59.2%, P*_trend_* = 0.88) remained stable over time. Outpatient referrals for follow-up increased slightly from 73.3% in 2007–2010 to 79.7% in 2015–2018 (P*_trend_* = 0.02), whereas neuroimaging use remained unchanged over time (37.3% in 2015–2018, P*_trend_* = 0.91).

As shown in Table 2, the use of all opioid analgesics dropped by nearly half or greater over time, while hydromorphone has remained the most commonly used opioid. The prescribing prevalence of the most widely used triptan, sumatriptan, was under 5% across the study period. The use of ergot alkaloids and other triptans was negligible among headache-related ED visits; thus, their national estimates were not reportable per NCHS’s reliability criteria. The most frequently used medication in the acetaminophen/NSAIDs group was ketorolac, which increased from 25.5% in 2007–2010 to 36.9% in 2015–2018 (P*_trend_* < 0.001). Among the antiemetics, metoclopramide (13.9% to 25.2%, P*_trend_* < 0.001) and ondansetron (14.0% to 18.6%, P*_trend_* < 0.001) use increased from 2007–2010 to 2015–2018, while promethazine use decreased by half (25.0% to 11.8%, P*_trend_* < 0.001), and prochlorperazine use remained stable (12.2% in 2015–2018, P*_trend_* = 0.52).

The most broadly used therapy among headache-related ED visits in 2007–2010 was an opioid with an antiemetic (21.0%), which decreased to 6.6% in 2015–2018 (Table 3)**.** Meanwhile, the combined use of acetaminophen/NSAIDs with antiemetic and diphenhydramine increased substantially from 3.9% to 15.7% and became the most prevalent therapy in 2015–2018. Opioid monotherapy use gradually decreased during the study period (8.8% to 1.9%). Table 4 shows the frequency of medications administered in EDs and prescribed at discharge by the medication group. The trends in medication use in EDs and at discharge were consistent with the findings from the overall trend analysis. Opioid analgesics were administered in EDs among 21.7% of headache-related ED visits and prescribed at discharge among 11.5% of headache-related ED visits in 2015–2018. Butalbital was administered in 2.0% of headache-related ED visits in 2015–2018 and prescribed at discharge in 5.3% of headache-related ED visits. Overall discharge medication use was infrequent among headache-related ED visits (e.g., ergot alkaloids/triptans: 2.4%; acetaminophen/NSAIDs: 12.8%; antiemetics: 12.2% in 2015–2018). Among headache-related ED visits, 85.5% of diphenhydramine use was coadministered with dopamine receptor antagonists, and 51.1% of dopamine receptor antagonist use was coadministered with diphenhydramine. When looking at the individual dopamine receptor antagonist use in headache-related ED visits, diphenhydramine coadministration accounted for 59.6%, 65.3%, 24.3% of metoclopramide, prochlorperazine and promethazine use, respectively (Figure S2).

Furthermore, stratified analyses by migraine versus NOS headache-related visits yielded similar trends as in the primary analysis (Figures S3 and S4). Compared to NOS headache-related visits, migraine-related visits had a greater use of ergot alkaloids/triptans (9.7% versus 1.9% in 2015–2018), antiemetics (80.3% versus 48.3%), diphenhydramine (47.5% versus 30.0%), and IV fluids (48.1% versus 37.9%). In addition, acetaminophen/ NSAID and corticosteroid use appeared to increase more rapidly among migraine-related visits. Neuroimaging use in NOS-headache-related visits was nearly twice that of migraine-related visits (44.3% versus 23.6% in 2015–2018).

### 3.3. Sensitivity Analyses

All the sensitivity analyses using different numbers of medication and/or diagnosis variables and with stricter exclusion criteria yielded similar findings with the primary analysis (Figures S5–S10).

## 4. Discussion

Using survey data that are nationally representative of the US population, our study yielded three important findings regarding medication and healthcare utilization for headache management in ED. First, the medication trends mostly reflected prescribing guidelines and policies. Opioid analgesic use in headache-related ED visits decreased by half over the 12-year study period, reflecting current AHS guidelines and current national opioid prescribing policies. Significant increases in acetaminophen/NSAIDs and corticosteroid use (1.4- to 2.3-fold) and the prevalent use of antiemetics (~60%) were also consistent with the AHS guideline recommendations. However, sumatriptan use remained low (<5%) over time in headache-related ED visits despite the AHS guideline recommendation. Second, nearly 40% of headache-related ED visits had neuroimaging ordered, and this pattern remained unchanged over time. Lastly, the majority (~80%) of headache-related ED visits had an outpatient referral for follow-up.

Migraine accounted for one-third of headache-related ED visits, and the subtype of headache was not determined in most of the remaining headache-related ED visits in our study. Consistent with prior studies using the same case definition from ED discharge diagnoses [28,37,38], the large proportions of NOS headaches (~64%) observed in our study may indicate the challenges of differentiating subtypes of primary headaches in ED settings because of a lack of comprehensive history for the patients’ headaches and the difficulty in applying ICHD-3 diagnostic criteria of primary headache disorders in ED settings. Vigano et al. reclassified most NOS headache diagnoses received in the ED as primary headache disorders when patients were re-examined in a headache unit [39]. A higher proportion of migraine (>60%) among headache-related ED visits was also reported in other studies using patient interviews, medical chart reviews, or neurologist consultations, which suggest the underdiagnosis of migraine and overestimation of NOS headaches in ED settings [40,41]. Many factors can contribute to the high proportion of the nonspecific diagnosis of headache in ED settings, including prioritizing acute symptomatic treatment and triaging those with life-threatening conditions over the precision of diagnosis in EDs, lack of knowledge of diagnostic criteria for primary headaches, and billing and coding practices.

The decreasing trend in opioid use in headache-related ED visits was compliant with the 2016 AHS guideline recommendations and the 2013 AHS Choosing Wisely Campaign [10,14]. A clinical policy released by the American College of Emergency Physicians (ACEP) in 2019 recommended nonopioid analgesics rather than opioids in the treatment of acute primary headaches in ED settings [42]. In addition to the AHS guidelines, an overall downward trend in opioid use in EDs during the last decade can largely be attributed to the multiple efforts to mitigate the opioid epidemic in the US [43,44]. In this study from 2007 to 2018, we observed a decrease in the proportion of opioid monotherapy among headache-related ED visits from 8.8% to 1.9%, which suggests a gradual shift in opioid prescribing patterns to use opioids as a last resort to treat headaches in EDs. However, our findings indicate that opioids were still used in more than one-quarter of headache-related ED visits between 2015 and 2018, which is consistent with findings from patients with migraines in other studies [27,45].

Aligned with the AHS guidelines [14] and general pain management recommendations [46,47,48], our findings showed an upward trend in nonopioid analgesic (i.e., acetaminophen/NSAIDs) use for headache-related ED visits, which offset the decline in opioid analgesic use. Our findings of the frequent use of antiemetics (~60%) in headache-related ED visits across years may reflect current evidence in the literature. The AHS guidelines particularly recommend intravenous metoclopramide and prochlorperazine among antiemetics, based on their noninferior or superior effects on migraine symptom relief compared to other acute migraine treatments (e.g., sumatriptan, octreotide, valproate) [49,50,51,52,53,54]. However, some discrepancies existed in the trend of antiemetic use for migraine treatment in EDs in previous studies due to the choice of antiemetic medications included in analysis. For example, Ruzek et al. focused on metoclopramide and prochlorperazine (both are dopamine receptor antagonists) and found that antiemetic use tripled (24% in 1999–2000 to 83% in 2014) among patients with migraine [45]. On the contrary, Mazer-Amirshahi et al. only examined promethazine and found its use remained stable (~24%) between 2001 and 2010 among headache-related ED visits [55]. Compared to previous studies, our study used a broader definition of antiemetics, including dopamine receptor antagonists and 5-HT3 receptor antagonists. Notably, metoclopramide use increased significantly, whereas promethazine use decreased proportionally, which accounts for the stability of the overall antiemetic use across the study period. The substantial decrease in promethazine use may reflect ED physicians’ awareness of an increased risk of serious tissue injuries associated with parenteral administration of promethazine due to its vesicant property [56].

Our study showed that diphenhydramine use in headache-related ED visits more than doubled from 2007–2010, and 85% of these ED visits with diphenhydramine use also concomitantly used dopamine receptor antagonists, despite AHS guidelines and current evidence against using diphenhydramine as a first-line treatment. A randomized controlled trial found that using diphenhydramine as adjuvant therapy among migraine patients treated with intravenous metoclopramide was neither superior to placebo for relieving migraine symptoms nor effective in preventing akathisia (i.e., an adverse effect of dopamine receptor antagonists) [57]. Other clinical trials also found a limited efficacy of diphenhydramine for preventing akathisia induced by metoclopramide [58,59], whereas existing evidence supports diphenhydramine use for the prevention of akathisia induced by intravenous prochlorperazine [60,61]. In addition, slow infusion of metoclopramide alone has been beneficial in preventing akathisia [62,63]. However, we observed similar coadministration patterns between prochlorperazine with diphenhydramine and metoclopramide with diphenhydramine. The substantial increasing trend in diphenhydramine use in our study could be attributed to its effect as a nonbenzodiazepine sedative-hypnotic. Nonetheless, diphenhydramine can worsen some dopamine receptor antagonists’ adverse effects, such as sedation and dizziness [60], and thus patients may require closer monitoring in ED settings when prescribed diphenhydramine. Finally, abuse potential or risk of dementia from prolonged use of anticholinergic drugs, including diphenhydramine, should not be overlooked [64,65].

Although the 2016 AHS guidelines recommend subcutaneous sumatriptan and parenteral dexamethasone use for acute migraine [14], we observed that triptans and corticosteroids were underutilized (<10%) in headache-related ED visits. Several factors may influence the underuse of triptans in EDs, including cardiovascular risks associated with triptans, a high incidence of adverse reactions related to sumatriptan injection, ED physicians’ unfamiliarity with injectable triptans, higher costs, and treatment failures with triptans prior to ED visits [66,67,68]. Furthermore, growing evidence supports dexamethasone use to prevent migraine recurrence within 24 to 72 h following ED discharge [14,69]. Therefore, identifying patients eligible for triptans and at high risk of headache recurrence (e.g., severe pain and nausea at baseline, presence of depression, and prolonged headaches) [70] for dexamethasone use may further improve patient outcomes of acute headache management in ED settings. Furthermore, most medications for headaches prescribed at discharge were prescribed infrequently (<13% in 2015–2018), with more than half of headache-related ED visits not receiving any medications. However, it is notable that opioid (11.5% in 2015–2018) and butalbital-containing medication use (5.3%) was more common than ergot alkaloid and triptan use (2.4%) at ED discharge. Butalbital-containing medications are approved for tension-type headache, but the current literature does not support their use in migraine [71]. Given the high risks for medication overuse headaches, intoxication, dependence, and withdrawal syndrome [71], butalbital-containing medication use at ED discharge should be limited. The 2019 ACEP clinical policy suggested that appropriately prescribing headache medications at discharge can reduce repeated ED visits for acute headaches. Further studies are needed to examine the barriers to prescribing medications for headaches at discharge [42].

In general, there has been continued growth in ED imaging utilization in the last couple of decades [72,73,74]. Despite current guidelines’ recommendations against neuroimaging use for patients with typical migraine headaches and a normal neurologic examination [9,10,11,12,13], ED physicians are more likely to order neuroimaging for patients with headaches than primary care physicians are, given that the top priority in headache management in the ED is to identify life-threatening secondary headaches. However, the overall proportion of pathological findings (e.g., stroke, SAH, central nervous system infections) was reported as low as approximately 2% of ED visits with a headache complaint [75]. In a prior NHAMCS study, neuroimaging use increased from 12.5% in 1998 to 31.0% in 2008 among atraumatic-headache-related ED visits [76]; however, in our study, neuroimaging use after 2008 remained stable without a further increase. Our finding may reflect ED physicians’ awareness of unnecessary neuroimaging for uncomplicated headaches, which exposes patients to radiation and imposes an economic burden on payers.

However, there is still much room for improvement in neuroimaging use for headaches in ED settings. Although there are society guidelines and recommendations, they do not always align. The 2019 ACEP clinical policy is focused on ruling out SAH, the most common malignant cause of secondary headache in EDs [42]. The American College of Radiology Appropriateness Criteria for headache rates differentiate imaging modalities depending on clinical scenarios [11], and the AHS evidence-based guideline for neuroimaging is centered around migraine, emphasizing the need for concerning signs/symptoms screening and establishing a specific diagnosis [12]. Our findings of a two-fold higher prevalence of neuroimaging orders in NOS-headache-related visits compared to migraine-related visits clearly indicates that neuroimaging use is highly related to uncertainty in the diagnostic work-up process. In a study by Sahai-Srivastava et al., patients with an NOS headache received head CT and lumbar puncture more often than those with an established specific diagnosis [77]. Fear of missing serious diagnoses with low probabilities and subsequent litigation is a commonly reported reason among ED physicians for unnecessary imaging orders in EDs [8]. Addressing expectations and concerns of patients, family members, and referring physicians, a busy practice when clinical evaluation is replaced by tests, discomfort with migraine as a diagnosis, and unfamiliarity with ICHD-3 diagnostic criteria can all contribute to overuse of neuroimaging in ED settings [12]. Future studies are warranted to develop a valid and reliable quick screening tool to identify patients with a primary headache that can be easily applied in ED settings.

Appropriate discharge care is important in headache management in EDs for two major reasons: (1) two-thirds of patients with headache-related visits do not receive a specific diagnosis at discharge and still need an appropriate diagnosis to be established, and (2) up to 80% of patients have a residual headache or headache recurrence within 24 h of ED discharge with persistent headache-related functional impairment if appropriate education and acute and preventive treatment are not implemented [70,78,79,80]. In our study, 73% of patients with headache-related ED visits were referred to an outpatient clinic or physician upon discharge. However, in many health care systems, outpatient appointments are difficult to obtain; thus, patients may continue relying on EDs for their chronic headache disorder management, even if they were referred for outpatient follow-up. Therefore, headache-related health outcomes of referrals upon discharge need to be further investigated.

Our study has several limitations, including the use of visit-level analyses that do not include patient follow-up data, a lack of reliable and validated algorithms for differentiating primary headaches from secondary headaches using diagnosis codes only, and a lack of information on the medication dose, route of administration, sequence of medication use, and specialty of consulting physicians. In addition, unmeasured confounders (e.g., regional or national drug shortages) might have influenced trends in medication use (e.g., meperidine) among headache-related ED visits over time. Despite these limitations, our study was the first to comprehensively examine medication and health service use for headache management in US EDs using nationally representative data.

## 5. Conclusions

Reflecting evidence-based guideline recommendations for headache management, opioid analgesic use substantially decreased from 2007 to 2018 among US headache-related ED visits. Nonopioid analgesic use and outpatient referrals for follow-up increased significantly, while ergot alkaloid/triptan use and visits with neuroimaging orders remained stable. Future studies are warranted to identify strategies to promote evidence-based treatments for headaches (e.g., sumatriptan and dexamethasone) and appropriate outpatient referrals for follow-up and to reduce unnecessary neuroimaging orders in EDs.

## Figures and Tables

**Table 1:** Patient characteristics among headache-related ED visits in the US: 2015 to 2018 NHAMCS data.

Weighted Visits	All Headaches	Migraine	NOS Headaches	
	10.2 Million (100.0%)	3.4 Million (32.9%)	6.6 Million (63.9%)	
Characteristics	Weighted %	Weighted %	Weighted %	SMD ^a^
Age				0.32
18–34	39.4	40.9	38.4	
35–49	31.3	37.2	28.1	
50–64	20.3	16.6	22.4	
≥65	9.0	5.3	11.1	
Sex				0.33
Female	72.9	82.0	68.0	
Male	27.1	18.0	32.0	
Race				0.41
White	70.3	82.2	64.7	
Non-White	29.7	17.8	35.3	
No. of chronic conditions				0.08
0	46.3	49.5	43.8	
1	26.3	24.6	27.0	
≥2	26.1	23.7	28.3	
Cardiovascular diseases ^b^	32.2	25.5	36.0	0.16
Depression	13.5	17.5	12.1	0.16
Pain scale				0.37
None (0)	5.0	3.0 *	6.1	
Mild (1–3)	4.9	2.7 *	6.2	
Moderate (4–6)	13.7	9.6	15.2	
Severe (7–10)	53.0	65.0	47.3	
Payment source				0.37
Commercial	31.3	35.7	29.2	
Medicare	16.3	13.7	17.2	
Medicaid	29.5	30.6	29.4	
Others	10.2	7.5	11.1	
No. of medications administered in ED				0.50
0	21.3	12.3	26.4	
1	10.5	5.2	13.3	
2	14.8	14.9	14.2	
≥3	53.5	67.6	46.1	
No. of medications prescribed at discharge				0.16
0	54.1	60.7	50.4	
1	21.1	17.2	22.7	
2	15.3	15.4	15.6	
≥3	9.5	6.6	11.3	
Provider type ^c^				
ED physician	85.7	86.6	85.5	0.02
Consulting physician	5.6	6.7	5.0	0.00
ED resident/intern	9.3	9.4	9.5	0.07
Nurse practitioner	10.3	8.9	10.9	0.00
Physician assistant	14.9	14.0	15.4	0.07
Geographic regions				0.09
South	38.3	33.7	40.2	
Northeast	14.4	14.5	14.3	
Midwest	24.1	27.7	22.4	
West	23.3	24.1	23.1	
Metropolitan area	86.2	81.2	88.5	0.23

Abbreviations: ED: emergency department; US: United States; NHAMCS: National Hospital Ambulatory Medical Care Survey; NOS: not otherwise specified; SMD: standardized mean difference; NCHS: National Center for Health Statistics. ^a^ SMD > 0.1 was considered as having a non-negligible difference between migraine-related visits and NOS-headache-related visits. ^b^ Cardiovascular diseases include cerebrovascular disease, history of stroke or transient ischemic attack, congestive heart failure, coronary artery disease, ischemic heart disease, history of myocardial infarction, hypertension, and hyperlipidemia. ^c^ Provider categories are not mutually exclusive. A patient can be seen by multiple providers during each ED visit. * The number of unweighted visits is fewer than 30 or the relative standard error is greater than 30. Weighted estimates of those values are considered unreliable by NCHS standards.

**Table 2:** Most frequently used medications among headache-related ED visits: 2007 to 2018 NHAMCS data.

Medication ^a^	2007–2010 (%)	2011–2014 (%)	2015–2018 (%)	P*_trend_* ^b^
Opioids	54.1	42.4	28.3	<0.001
Codeine	1.0	0.8 *	3.6	<0.001
Hydrocodone	16.0	12.4	6.6	<0.001
Hydromorphone	17.3	14.9	8.8	<0.001
Meperidine	6.6	2.2	0.9 *	<0.001
Morphine	9.3	9.8	5.1	<0.001
Nalbuphine	4.2	1.1	1.2 *	<0.001
Oxycodone	6.7	7.1	3.0	<0.001
Butalbital	5.1	6.5	6.4	0.22
Ergot alkaloids/Triptans	5.2	5.3	4.7	0.88
Sumatriptan	4.3	4.8	3.8	0.52
Acetaminophen/NSAIDs	37.2	45.7	52.4	<0.001
Acetaminophen	6.7	12.2	12.2	<0.001
Ibuprofen	9.2	10.3	10.3	0.68
Ketorolac	25.5	36.9	36.9	<0.001
Naproxen	1.8	2.4	2.3	0.51
Antiemetics	59.3	57.7	59.2	0.88
Dopamine receptor antagonists	27.5	28.8	38.0	<0.001
Metoclopramide	13.9	19.8	25.2	<0.001
Prochlorperazine	13.4	7.6	12.2	<0.001
Promethazine	25.0	15.4	11.8	<0.001
5-HT3 antagonists	14.0	24.1	18.6	<0.001
Ondansetron	14.0	24.1	18.6	<0.001
Diphenhydramine	16.5	24.0	35.8	<0.001
Corticosteroids	2.7	3.6	6.2	<0.001
Dexamethasone	0.7 *	1.6	3.5	<0.001
Methylprednisolone	1.4	1.3	1.8 *	0.60

Abbreviations: ED: emergency department; NHAMCS: National Hospital Ambulatory Medical Care Survey; NSAIDs: nonsteroidal anti-inflammatory drugs; NCHS: National Center for Health Statistics. ^a^ Medications were administered in the ED or prescribed at ED discharge. We presented all medications in each group that meet the NCHS’s reliability criteria for at least 2 out of 3 values in the year categories. Among the medications in Table S2, the use of those medications not listed here is negligible. ^b^ All P*_trend_* were adjusted for age, sex, race, payment source, and practice region. * The number of unweighted visits is fewer than 30 or the relative standard error is greater than 30. Weighted estimates of those values are considered unreliable by NCHS standards.

**Table 3:** Most common therapies administered among headache-related ED visits: 2007 to 2018 NHAMCS data.

Most Common Therapies	2007–2010 (%)	2011–2014 (%)	2015–2018 (%)
Acetaminophen/NSAIDs	8.9	8.8	10.6
Acetaminophen/NSAIDs + Antiemetic	7.4	8.8	7.6
Acetaminophen/NSAIDs + Antiemetic + Diphenhydramine	3.9	7.0	15.7
Antiemetic	4.8	4.2	3.2
Antiemetic + Diphenhydramine	4.9	4.9	7.4
Opioid	8.8	5.0	1.9
Opioid + Acetaminophen/NSAIDs + Antiemetic	5.1	4.5	3.8
Opioid + Antiemetic	21.0	13.7	6.6
Opioid + Antiemetic + Diphenhydramine	2.9	3.4	2.1

Abbreviations: ED: emergency department; NHAMCS: National Hospital Ambulatory Medical Care Survey; NSAIDs: nonsteroidal anti-inflammatory drugs.

**Table 4:** Frequency of medication use stratified by those administered in ED and prescribed at discharge among headache-related ED visits in the US: NHAMCS data for 2007–2010 and 2015–2018.

Medications	2007–2010		2015–2018	
	Administered in ED (%)	Prescribed at Discharge (%)	Administered in ED (%)	Prescribed at Discharge (%)
Opioids	44.0	23.2	21.7	11.5
Butalbital	1.1 *	4.0	2.0	5.3
Acetaminophen/NSAIDs	30.1	11.9	47.9	12.8
Ergot alkaloids/Triptans	3.1 *	3.6 *	2.8	2.4
Antiemetics	55.5	11.6	56.2	12.2
Diphenhydramine	15.7	1.2	35.0	2.2
Corticosteroids	2.3	0.3 *	5.0	1.5 *

Abbreviations: ED: Emergency Department; US: United States; NHAMCS: National Hospital Ambulatory Medical Care Survey; NSAID: nonsteroidal anti-inflammatory drug; NCHS: National Center for Health Statistics. * The number of unweighted visits is fewer than 30 or the relative standard error is greater than 30. Weighted estimates of those values are considered unreliable by NCHS standards.

## Data Availability

The data presented in this study are openly available from the National Center for Health Statistics at https://www.cdc.gov/nchs/ahcd/datasets_documentation_related.htm (accessed on 30 November 2021).

## Supplementary Materials

The following are available online at https://www.mdpi.com/article/10.3390/jcm11051401/s1, Table S1: ICD-9-CM/ICD-10-CM codes used to identify headache-related ED visits in NHAMCS, Table S2: Multum Lexicon Plus^®^ generic codes used to identify medication use among headache-related ED visits in NHAMCS, Table S3: ICD-9-CM/ICD-10-CM codes used to identify medical conditions associated with secondary headaches, Figure S1: Number and proportion of headache-related ED visits in the US: 2007 to 2018 NHAMCS data (primary analysis and sensitivity analysis 1), Figure S2: Diphenhydramine coadministration among headache-related ED visits with dopamine receptor antagonist administration: 2007 to 2018 NHAMCS data, Figure S3: Trends in medication use, neuroimaging use, and outpatient referral for follow-up among migraine-related ED visits: 2007 to 2018 NHAMCS data (primary analysis), Figure S4: Trends in medication use, neuroimaging use, and outpatient referral for follow-up among not-otherwise-specified headache-related ED visits: 2007 to 2018 NHAMCS data (primary analysis), Figure S5: Number and proportion of headache-related ED visits in the US: 2007 to 2018 NHAMCS data (sensitivity analyses 2 and 3), Figure S6: Number and proportion of headache-related ED visits in the US: 2007 to 2018 NHAMCS data (sensitivity analysis 4), Figure S7: Trends in medication use, neuroimaging use, and referrals to follow-up among headache-related ED visits: 2007 to 2018 NHAMCS data (sensitivity analysis 1), Figure S8: Trends in medication use, neuroimaging use, and referrals to follow-up among headache-related ED visits: 2007 to 2018 NHAMCS data (sensitivity analysis 2), Figure S9: Trends in medication use, neuroimaging use, and referrals to follow-up among headache-related ED visits: 2007 to 2018 NHAMCS data (Sensitivity analysis 3), Figure S10: Trends in medication use, neuroimaging use, and referrals to follow-up among headache-related ED visits: 2007 to 2018 NHAMCS data (sensitivity analysis 4).
