# Supplementary material for: Trends in the Management of Headache Disorders in US Emergency Departments: Analysis of 2007–2018 National Hospital Ambulatory Medical Care Survey Data
Source: J Clin Med. 2022 Mar 3;11(5):1401. doi: 10.3390/jcm11051401 (PMC8910868; doi:10.3390/jcm11051401)
Supplement: Supplementary file 1 [file jcm-11-01401-s001.zip › jcm-1511343-supplementary.pdf]

SUPPLEMENTARY MATERIALS

Table S1. ICD-9-CM/ICD-10-CM codes used to identify headache-related ED visits in NHAMCS

Table S2. Multum Lexicon Plus® generic codes used to identify medication use among headache-related ED visits in NHAMCS

Table S3. ICD-9-CM/ICD-10-CM codes used to identify medical conditions associated with secondary headaches

Figure S1. Number and proportion of headache-related ED visits in the US: 2007 to 2018 NHAMCS data (Primary analysis & sensitivity analysis 1)

Figure S2. Diphenhydramine co-administration among headache-related ED visits with dopamine receptor antagonist administration: 2007 to 2018 NHAMCS data

Figure S3. Trends in medication use, neuroimaging use, and outpatient referral for follow-up among migraine-related ED visits: 2007 to 2018 NHAMCS data (Primary analysis)

Figure S4. Trends in medication use, neuroimaging use, and outpatient referral for follow-up among not-otherwise-specified headache-related ED visits: 2007 to 2018 NHAMCS data (Primary analysis)

Figure S5. Number and proportion of headache-related ED visits in the US: 2007 to 2018 NHAMCS data (Sensitivity analyses 2 & 3)

Figure S6. Number and proportion of headache-related ED visits in the US: 2007 to 2018 NHAMCS data (Sensitivity analysis 4)

Figure S7. Trends in medication use, neuroimaging use, and referrals to follow-up among headache-related ED visits: 2007 to 2018 NHAMCS data (Sensitivity analysis 1)

Figure S8. Trends in medication use, neuroimaging use, and referrals to follow-up among headache-related ED visits: 2007 to 2018 NHAMCS data (Sensitivity analysis 2)

Figure S9. Trends in medication use, neuroimaging use, and referrals to follow-up among headache-related ED visits: 2007 to 2018 NHAMCS data (Sensitivity analysis 3)

Figure S10. Trends in medication use, neuroimaging use, and referrals to follow-up among headache-related ED visits: 2007 to 2018 NHAMCS data (Sensitivity analysis 4)

Table S1. ICD-9-CM/ICD-10-CM codes used to identify headache-related ED visits in NHAMCS

Headache subtypes	ICD-9-CM Codes	ICD-10-CM Codes
Migraine	346.xx	G43.xxx
Tension-type headache	339.1x 307.81	G44.2xx
Trigeminal autonomic cephalgia	339.0x	G44.0xx
NOS headache	784.0	R51.x

Abbreviations: ED: emergency department; NHAMCS: National Hospital Ambulatory Medical Care Survey; ICD-9-CM: International Classification of Diseases, Ninth Revision, Clinical Modification; ICD-10-CM: International Classification of Diseases, Tenth Revision, Clinical Modification; NOS: Not Otherwise Specified

Table S2. Multum Lexicon Plus® generic codes used to identify medication use among headache-related ED visits in NHAMCS

Medication Class and Drugs	Generic Codes
Opioids[a]	
Buprenorphine	d00840
Butorphanol	d00838
Codeine	d00012 d03423 d03425 d03426
Fentanyl	d00233
Hydrocodone	d03075 d03428† d04225†
Hydromorphone	d00255
Meperidine	d00017
Morphine	d00308
Nalbuphine	d00839
Oxycodone	d00329 d03431†
Propoxyphene	d00360 d03434† d03435†
Tapentadol	d07453
Tramadol	d03826 d04766
Butalbital	d03061 d03455† d03456† d03457†
Ergot alkaloids/Triptans	
Dihydroergotamine	d00211
Ergotamine	d00222 d04120
Almotriptan	d04757
Eletriptan	d04849
Frovatriptan	d04776
Rizatriptan	d04328
Sumatriptan	d03160 d07130†
Zolmitriptan	d04253
Acetaminophen/NSAIDs	
Acetaminophen	a11027 d00049 d03297 d03445† d05701 d03439
Aspirin	a10464 a11190
Diclofenac	d00848
Etodolac	d00851
Flurbiprofen	d00239
Ibuprofen	d00015
Indomethacin	d00039
Ketorolac	d00273
Mefenamic Acid	d00285
Meloxicam	d04532
Nabumetone	d00310
Naproxen	d00019
Phenylbutazone	d00341
Antiemetics[b]	
Dopamine receptor antagonists	
Chlorpromazine	d00064
Prochlorperazine	d00355
Droperidol	d00219
Haloperidol	d00027
Metoclopramide	d00298
Domperidone	a59812
Promethazine	d00787

5-HT3 receptor antagonists	
Ondansetron	d00867
Granisetron	d03171
Dolasetron	d04214
Diphenhydramine	d00212
Corticosteroids	
Dexamethasone	d00206
Methylprednisolone	d00293
Prednisone	d00350

Abbreviations: ED: Emergency Department; NHAMCS: National Hospital Ambulatory Medical Care Survey; NSAID: Non-Steroidal Anti-Inflammatory Drug

[a] Opioid antitussives classified as '124: antitussives' or '132: upper respiratory combinations' by Multum Lexicon Plus® were excluded.

[b] Antiemetic class includes dopamine receptor antagonists and 5-HT3 antagonists. Although promethazine has both antihistamine and antidopaminergic properties, it is classified as a dopamine receptor antagonist. Because it is commonly used for antiemetic purpose, rather than preventing akathisia such as diphenhydramine.

† These medications are combination products. We used the following hierarchy order based on their analgesic potency to classify them: opioids > butalbital > ergot alkaloids/triptans > acetaminophen/NSAIDs > diphenhydramine.

Table S3. ICD-9-CM/ICD-10-CM codes used to identify medical conditions associated with secondary headaches

Conditions	ICD-9-CM Codes	ICD-10-CM Codes
Acute angle closure glaucoma	365.22	H40.21
Benign intracranial hypertension	348.2	G93.2
Brain tumor	239.6, 237.5	D43.0 D43.1 D43.2 D49.6
Carbon monoxide poisoning	986	T58.xx
Carotid artery dissection	443.21	I77.71
Cerebral aneurysm	437.3	I67.1
Cerebral infarction	434.xx	I63.xx
Cerebral venous thrombosis	437.6	I67.6
Chronic sinusitis	473.x	J32.x
Complicated headache syndromes	339.4x	G44.5x
Drug-induced headache	339.3	G44.4x
Encephalitis	323.xx 062.x 063.x 064	G04.xx G05.xx A83.x A84.xx A85.x A86
Giant cell arteritis	446.5	M31.6
Hypertension encephalopathy	437.2	I67.4
Intracerebral hemorrhage	431.xx	I61.xx
Intracranial hemorrhage	432.xx	I62.xx
Intracranial space-occupying lesions	784.2	R90.0
Malignant neoplasm of brain	191.x	C71.x
Meningitis	320.xx 321.xx 322.xx	G00.xx G01.xx G02.xx G03.xx A87.x
Occipital neuralgia	723.8	M54.81
Other specified headache syndromes	339.8x	G44.8x
Post-traumatic headache	339.2x	G44.3x
Preeclampsia	642.4x 642.5x 642.6x 642.7x	O11.x O14.xx
Reversible cerebral vasoconstriction syndrome	435.9	I67.841
Subarachnoid hemorrhage	430.xx	I60.xx
Subdural hematoma	432.1	I62.x
Tolosa-hunt syndrome	378.55	H49.4x
Traumatic brain injury	800.xx 801.xx 802.xx 803.xx 804.xx 850.xx 851.xx 852.xx 853.xx 854.xx	S00.xx S01.xx S02.x S03.xx S04.xx S05.xx S06.x S07.x S08.xx S09.xx
Vertebral artery dissection	443.24	I77.74

Abbreviations: ICD-9-CM: International Classification of Diseases, Ninth Revision, Clinical Modification; ICD-10-CM: International Classification of Diseases, Tenth Revision, Clinical Modification

Figure S1. Number and proportion of headache-related ED visits in the US: 2007 to 2018 NHAMCS data (Primary analysis & sensitivity analysis 1)

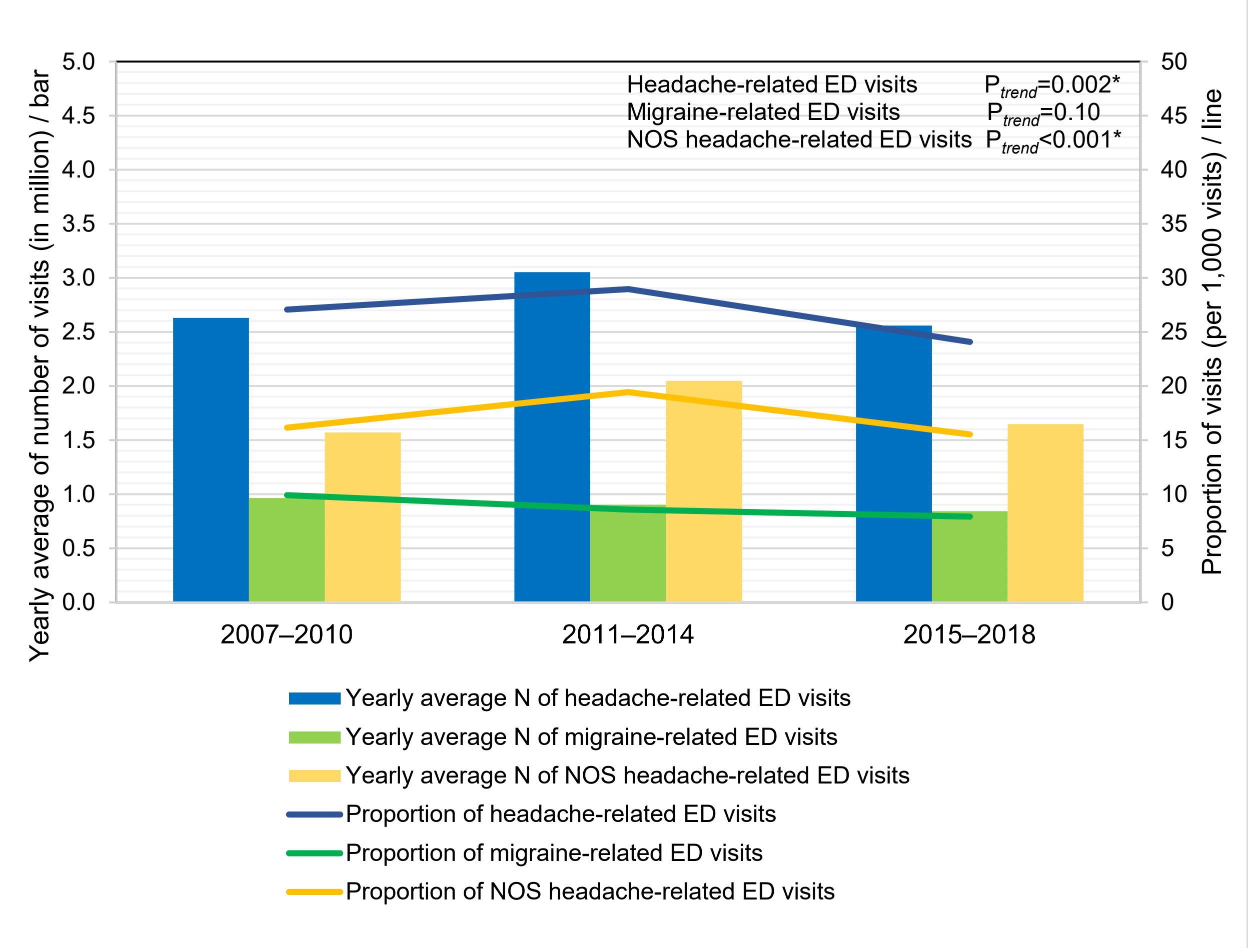

Abbreviations: ED: Emergency Department; US: United States; NHAMCS: National Hospital Ambulatory Medical Care Survey; NOS: Not Otherwise Specified

* A statistically significant trend with P_{trend}<0.001. All P_{trend} were adjusted for age, sex, race, payment source, and practice region.

Figure S2. Diphenhydramine co-administration among headache-related ED visits with dopamine receptor antagonist administration: 2007 to 2018 NHAMCS data

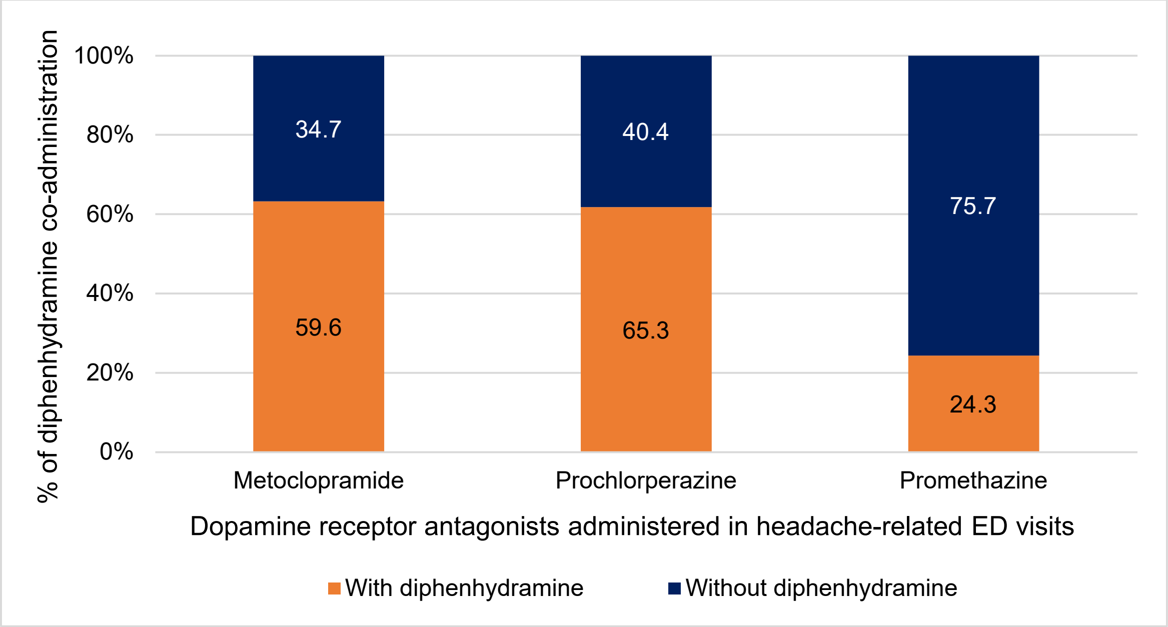

Figure S3. Trends in medication use, neuroimaging use, and outpatient referral for follow-up among migraine-related ED visits: 2007 to 2018 NHAMCS data (Primary analysis)

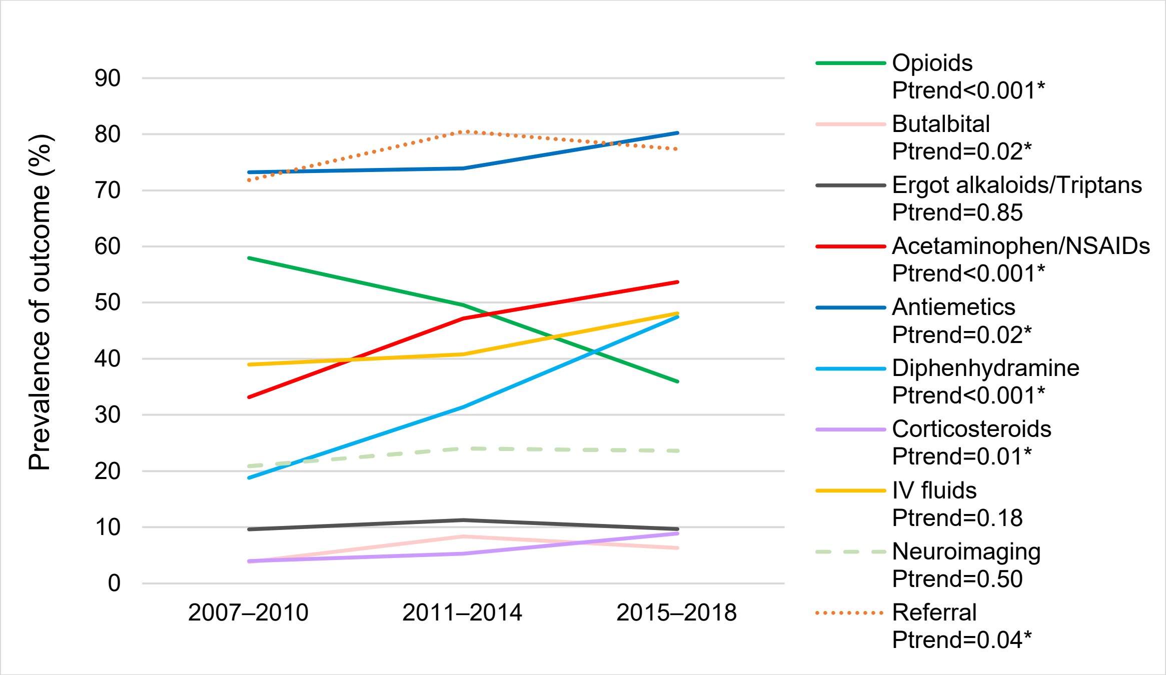

Abbreviations: ED: Emergency Department; NHAMCS: National Hospital Ambulatory Medical Care Survey; NSAID: Non-Steroidal Anti-Inflammatory Drug

* A statistically significant trend with P_{trend}<0.001. All P_{trend} were adjusted for age, sex, race, payment source, and practice region.

Figure S4. Trends in medication use, neuroimaging use, and outpatient referral for follow-up among not-otherwise-specified headache-related ED visits: 2007 to 2018 NHAMCS data (Primary analysis)

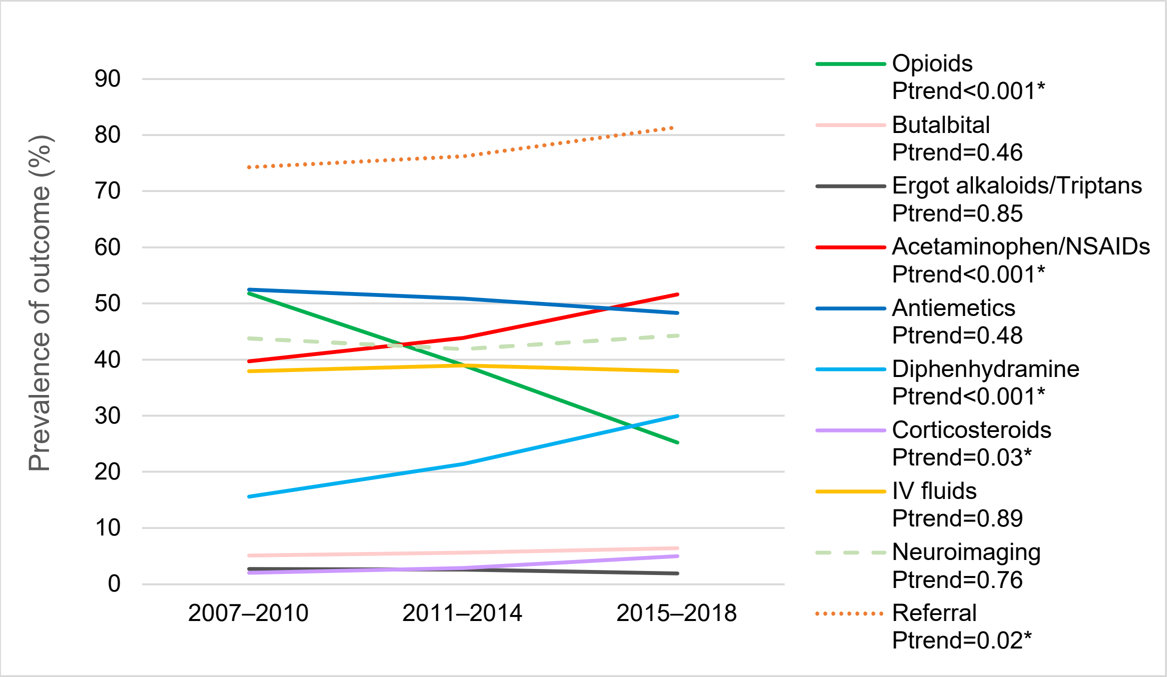

Abbreviations: ED: Emergency Department; NHAMCS: National Hospital Ambulatory Medical Care Survey; NSAID: Non-Steroidal Anti-Inflammatory Drug

* A statistically significant trend with P_{trend}<0.001. All P_{trend} were adjusted for age, sex, race, payment source, and practice region.

Figure S5. Number and proportion of headache-related ED visits in the US: 2007 to 2018 NHAMCS data (Sensitivity analyses 2 & 3)

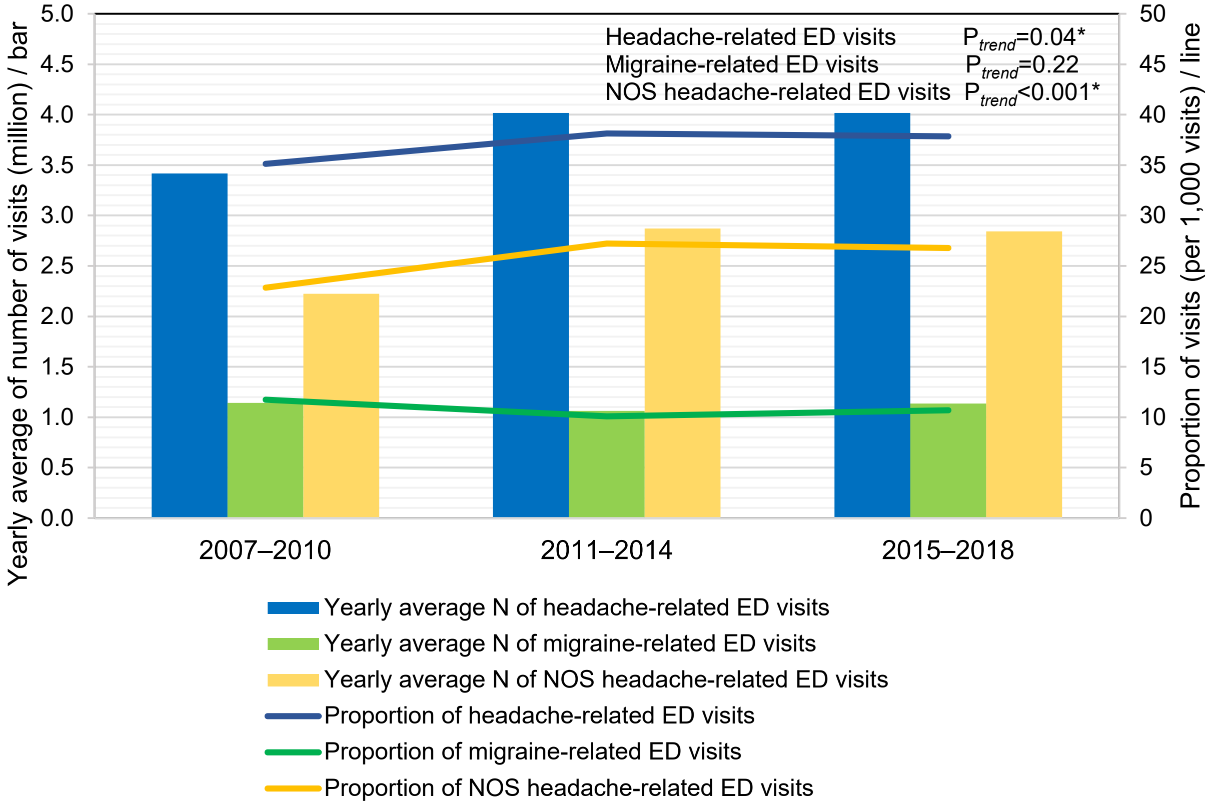

Abbreviations: ED: Emergency Department; US: United States; NHAMCS: National Hospital Ambulatory Medical Care Survey; NOS: Not Otherwise Specified

* A statistically significant trend with P_{trend}<0.001. All P_{trend} were adjusted for age, sex, race, payment source, and practice region.

Figure S6. Number and proportion of headache-related ED visits in the US: 2007 to 2018 NHAMCS data (Sensitivity analysis 4)

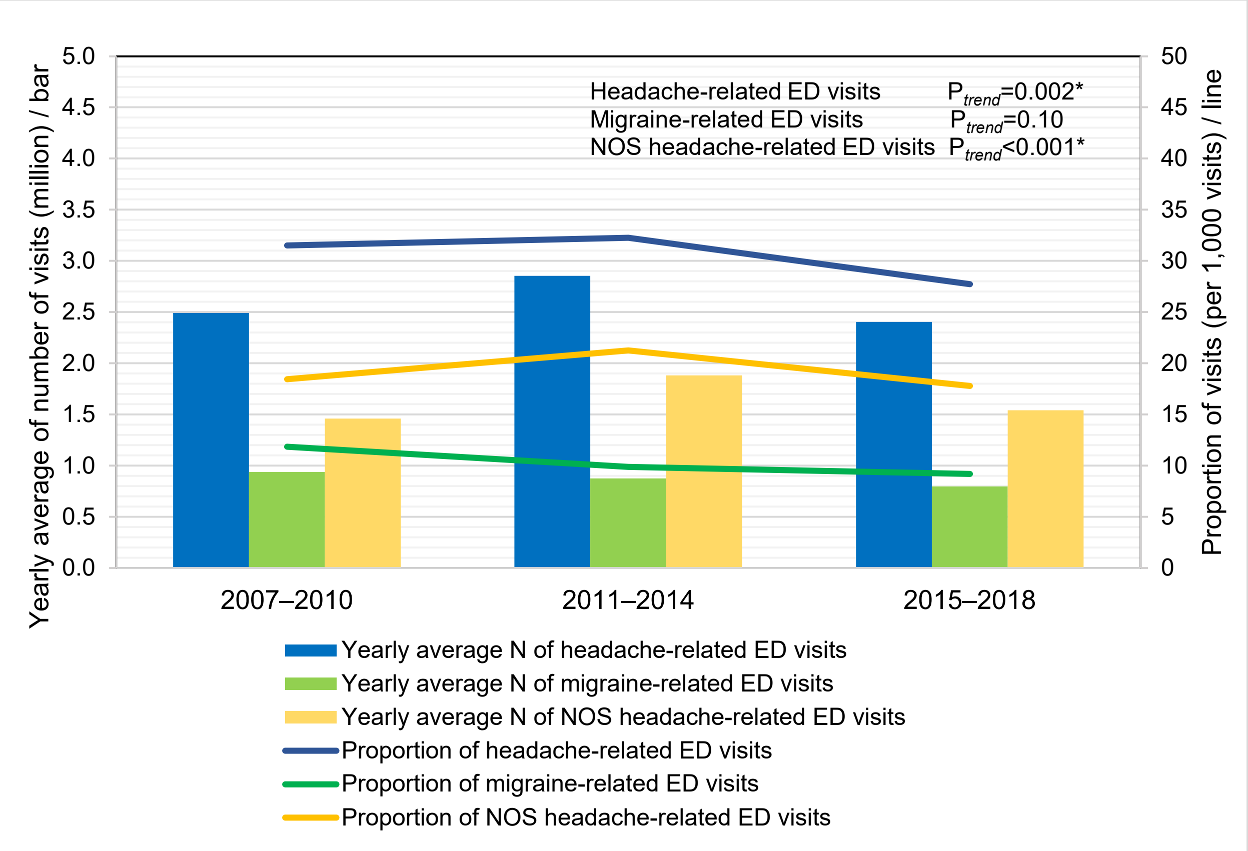

Abbreviations: ED: Emergency Department; US: United States; NHAMCS: National Hospital Ambulatory Medical Care Survey; NOS: Not Otherwise Specified

* A statistically significant trend with P_{trend}<0.001. All P_{trend} were adjusted for age, sex, race, payment source, and practice region.

Figure S7. Trends in medication use, neuroimaging use, and referrals to follow-up among headache-related ED visits: 2007 to 2018 NHAMCS data (Sensitivity analysis 1)

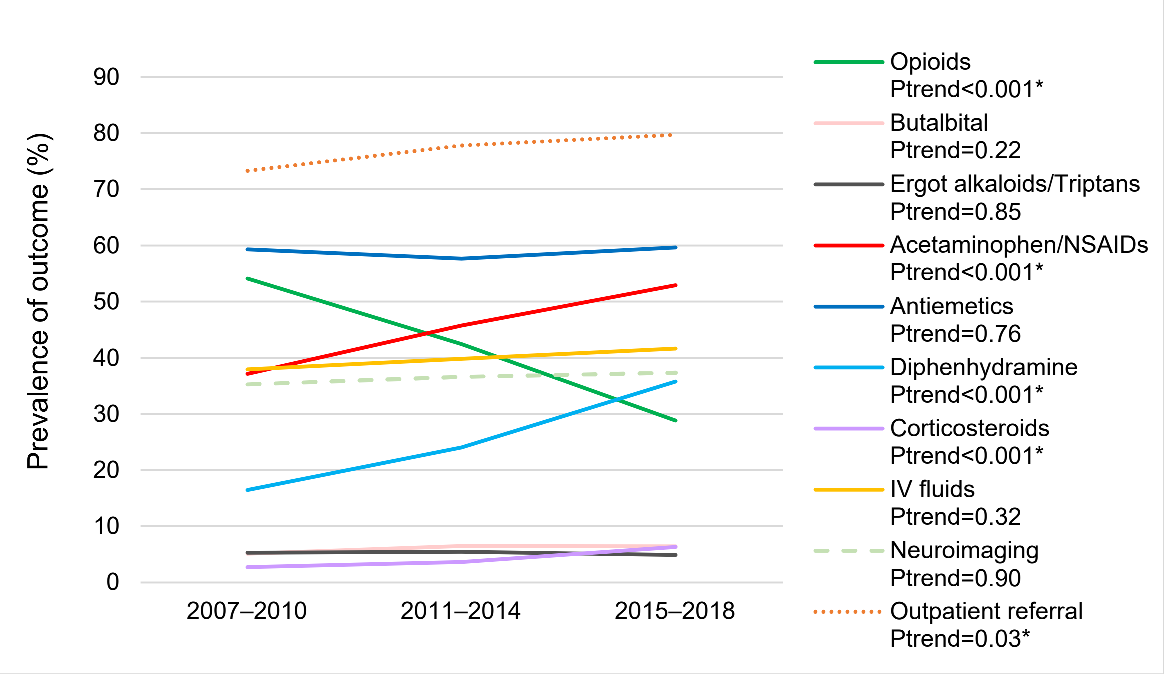

Abbreviations: ED: Emergency Department; NHAMCS: National Hospital Ambulatory Medical Care Survey; NSAID: Non-Steroidal Anti-Inflammatory Drug

* A statistically significant trend with P_{trend}<0.001. All P_{trend} were adjusted for age, sex, race, payment source, and practice region.

Figure S8. Trends in medication use, neuroimaging use, and referrals to follow-up among headache-related ED visits: 2007 to 2018 NHAMCS data (Sensitivity analysis 2)

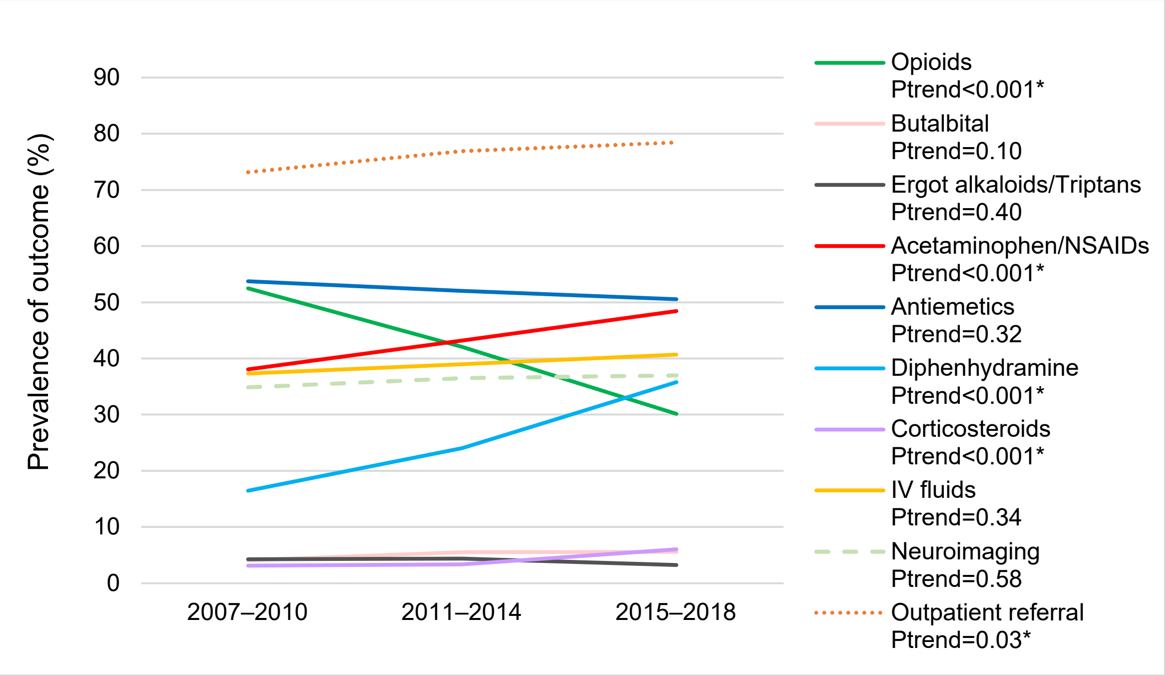

Abbreviations: ED: Emergency Department; NHAMCS: National Hospital Ambulatory Medical Care Survey; NSAID: Non-Steroidal Anti-Inflammatory Drug
* A statistically significant trend with P_{trend}<0.001. All P_{trend} were adjusted for age, sex, race, payment source, and practice region.

Figure S9. Trends in medication use, neuroimaging use, and referrals to follow-up among headache-related ED visits: 2007 to 2018 NHAMCS data (Sensitivity analysis 3)

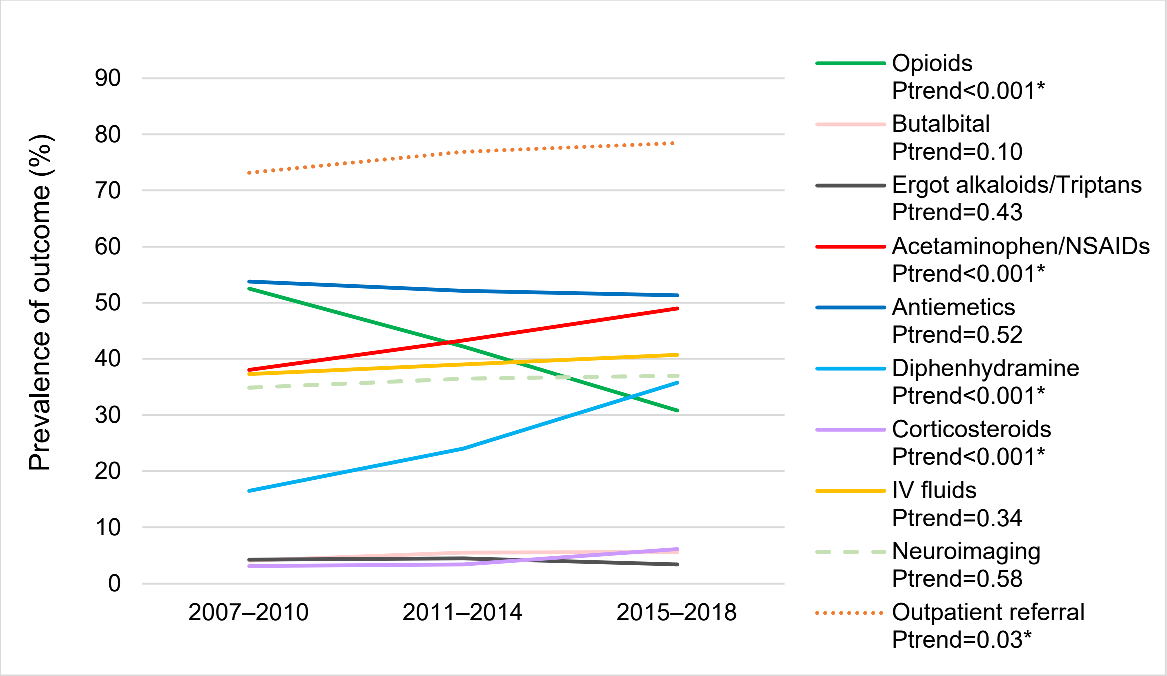

Abbreviations: ED: Emergency Department; NHAMCS: National Hospital Ambulatory Medical Care Survey; NSAID: Non-Steroidal Anti-Inflammatory Drug

* A statistically significant trend with P_{trend}<0.001. All P_{trend} were adjusted for age, sex, race, payment source, and practice region.

Figure S10. Trends in medication use, neuroimaging use, and referrals to follow-up among headache-related ED visits: 2007 to 2018 NHAMCS data (Sensitivity analysis 4)

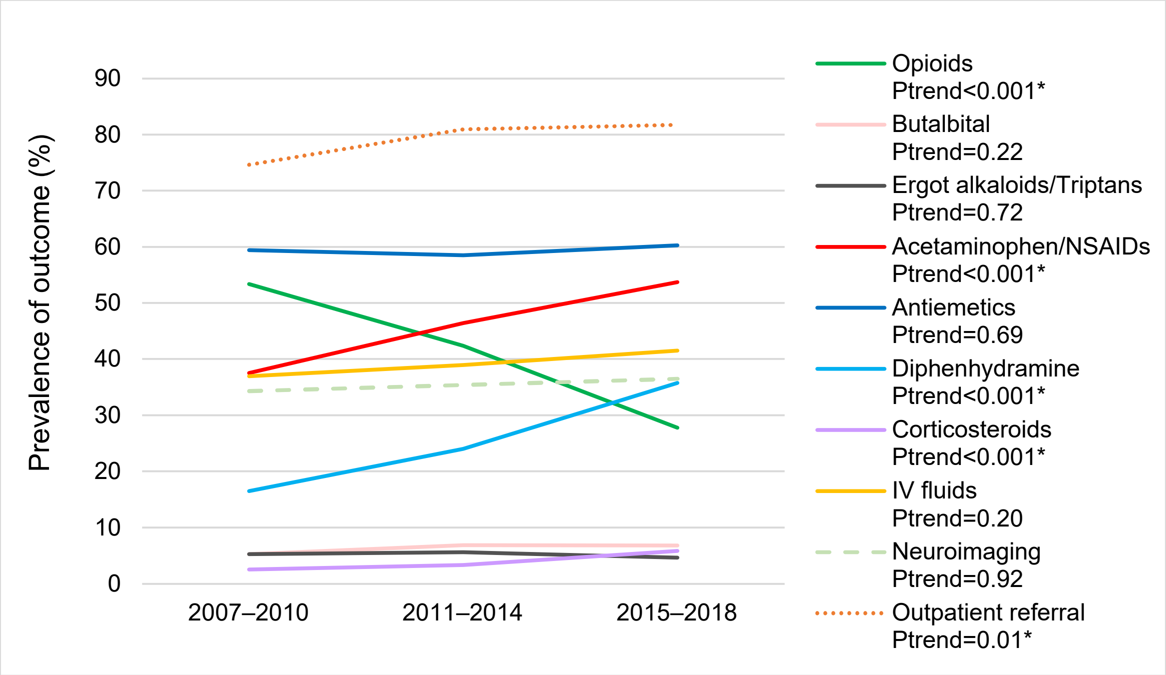

Abbreviations: ED: Emergency Department; NHAMCS: National Hospital Ambulatory Medical Care Survey; NSAID: Non-Steroidal Anti-Inflammatory Drug

* A statistically significant trend with P_{trend}<0.001. All P_{trend} were adjusted for age, sex, race, payment source, and practice region.
